# Therapeutic implications of PD-L1 expression in bladder cancer with squamous differentiation

**DOI:** 10.1186/s12885-020-06727-2

**Published:** 2020-03-18

**Authors:** Ronja Morsch, Michael Rose, Angela Maurer, Maria Angela Cassataro, Till Braunschweig, Ruth Knüchel, Thomas-Alexander Vögeli, Thorsten Ecke, Markus Eckstein, Veronika Weyerer, Irene Esposito, Maximilian Ackermann, Günter Niegisch, Nadine T. Gaisa, on behalf of the German Study Group of Bladder Cancer (DFBK e.V.), Michael Rose, Ruth Knüchel, Thorsten Ecke, Nadine T. Gaisa

**Affiliations:** 1 Department of Urology, University Hospital RWTH Aachen University, Aachen, Germany; 2 Institute of Pathology, University Hospital RWTH Aachen University, Aachen, Germany; 3 Department of Urology, HELIOS Hospital, Bad Saarow, Germany; 4 Institute of Pathology, University Hospital Erlangen, Friedrich-Alexander University Erlangen-Nürnberg, Erlangen, Germany; 5 Institute of Pathology, Heinrich Heine University Düsseldorf, Düsseldorf, Germany; 6 Department of Urology, Heinrich Heine University Düsseldorf, Düsseldorf, Germany

**Keywords:** PD-L1, Immunotherapy, Bladder cancer, Squamous cell carcinoma

## Abstract

**Background:**

Immune checkpoint inhibitors (ICI) are an integral part of bladder cancer therapy, however, the relevance of ICI treatment for mixed and pure squamous cell carcinoma of the bladder remains poorly studied. Therefore, we analysed the expression of programmed death-ligand 1 (PD-L1) in urothelial carcinomas with squamous differentiation (UC/SCC) and pure squamous cell carcinoma (SCC) of the bladder and studied a UC/SCC patient with ICI therapy.

**Methods:**

Tissue microarrays of 45 UC/SCC and 63 SCC samples were immunohistochemically stained with four anti-PD-L1 antibodies (28–8, 22C3, SP142 and SP263). PD-L1 expression was determined for tumour cells (TP-Score), immune cells (IC-Score) and combined (CPS, combined positive score). In addition, we present clinical and histological data of an UC/SCC patient with nivolumab therapy.

**Results:**

Overall, positive PD-L1 staining ranged between 4.8 and 61.9% for IC and 0 and 51.2% for TC depending on the used antibody. There were no significant differences between UC/SCC and SCC. According to current FDA guidelines for example for first line therapy of urothelial cancer with pembrolizumab (CPS ≥ 10), a subset of SCC patients up to 20% would be eligible. Finally, our UC/SCC index patient revealed excellent therapy response regarding his lung metastasis.

**Conclusions:**

Our data reveal a PD-L1 expression in squamous differentiated carcinomas comparable with current data shown for urothelial tumours. In accordance with the encouraging clinical data of the index patient we suggest ICI treatment also for mixed and pure SCC of the urinary bladder.

## Background

The immune system plays an important role in disease protection and cell clearing by orchestrating T-cell mediated immune responses [1]. Several immune checkpoints ensure correct cell recognition. Under normal conditions programmed cell death-1 (PD-1)-receptor is expressed on the surface of activated T-cells and its ligand programmed cell death ligand-1 (PD-L1) on the surface of dendritic cells and macrophages. PD1/PD-L1 interaction induces the activation of Src homology region 2 domain-containing phosphatases modulating the T-cell antigen receptor (TCR) signalling and mediating immune tolerance to self-antigens [2, 3]. However, cancer cells can misuse these checkpoints by overexpressing PD-L1 in tumour cells protecting themselves from cytotoxic T-cell immune detection and elimination [4]. Recently, immunotherapy targeting the PD1/PD-L1 axis has emerged as promising field in anti-cancer therapy for various tumour entities (including non-small lung cancer, renal cell cancer, or head and neck squamous cell cancer) [5]. By blocking theses immune checkpoint proteins, cancer cells’ resistance to immune response can be overcome and effective T-cell response against cancer cells can be restored [4, 6]. Meanwhile ICI treatment is an integral part of first line (in platinum ineligible patients) and second line clinical management of patients with urothelial carcinoma: Five different checkpoint inhibitors, i.e. pembrolizumab, nivolumab, atezolizumab, durvalumab, and avelumab, have been assessed in clinical trials of advanced bladder cancer during the last years [7] and can be used for second line treatment, but only pembrolizumab and atezolizumab are currently approved by the Food and Drug Administration (FDA) and the European Medicines Agency (EMA) for first line therapy in urothelial cancer [8, 9]. In this setting treatment with ICIs depends on complementary PD-L1 assessment based on different PD-L1 antibodies and immunohistochemical assays creating a wealth of different scoring algorithms and evaluation criteria. Recent studies revealed substantial inter-assay heterogeneity of PD-L1 expression in different tumour entities including bladder cancer with also some degree of inter-observer diversity as well [10–12].

The impact of ICI treatment in patients with rare bladder tumours remains poorly studied. Histologically, bladder cancer comprises a heterogeneous group of tumours including those with squamous differentiation (SD-BLCA), i.e. urothelial cancers with squamous differentiation (UC/SCC) and pure squamous cell carcinoma (SCC). SD-BLCA is characterized by poor outcome and lack of effective (neo) adjuvant therapy [13–15]. Pure SCC can be classified into two subgroups, i.e. SCC associated with schistosomiasis whose incidence rate is increased in regions where schistosomiasis is endemic (e.g. in the Middle East), and non-Schistosomiasis associated SCC [16]. Recently PD-L1 expression was studied in Schistosomiasis-related SCC of the bladder highlighting an association between negative PD-L1 expression and clinico-pathological parameters like tumour stage and unfavourable patients’ outcome [17]. In 2018 Udager and colleagues analysed PD-L1 protein expression in 17 pure SCC samples of the urinary bladder demonstrating frequent PD-L1 positivity (65%) [18]. Reis et al. confirmed strong PD-L1 expression in immune and tumour cells in 16 urothelial cancers with squamous differentiation [19], however, a comprehensive study involving the most prominent diagnostic PD-L1 antibodies and corresponding scoring algorithms (immune cell (IC)-score, tumour proportion (TP)-score and combined positivity score (CPS)) in non-Schistosomiasis SCC is still missing. Therefore, we aim to give insights into the therapeutic implications of PD-L1 expression in non-Schistosomiasis associated SD-BLCA by assessing PD-L1 expression using four different PD-L1 antibodies (DAKO 28–8, DAKO 22C3, Ventana SP263, Ventana SP142) in both a retrospective cohort including 45 mixed UC/SCC / 63 pure SCC and in tissue samples derived from a SD-BLCA index patient who showed excellent response of pulmonary metastasis upon nivolumab treatment.

## Methods

### Patient samples and tissue microarray construction

Formalin-fixed paraffin-embedded (FFPE) samples of primary non-Schistosomiasis-related SCC and mixed UC/SCC (urothelial bladder cancer with substantial squamous components > 50% of tumour area) were collected from collaborating Institutes of Pathology in Germany and the German Study Group of Bladder Cancers (DFBK e.V.). Tissue microarrays (TMA) with a minimum of two cores from different tumour areas of FFPE samples (45 UC/SCC, 63 SCC) were constructed. For the index patient, whole tissue slides were used for analysis. The patient consented the use of his tissue samples stored at the biobank of the Comprehensive Cancer Centre Düsseldorf and the according clinical data (IRB approval: number 4601; April 16th 2014). The retrospective anonymous study was approved by the local ethics committee (RWTH EK 009/12).

### Immunohistochemistry

FFPE slides were stained for protein expression of programmed death-ligand 1 (PD-L1) with four different antibodies [28–8 (Agilent/DAKO, California, USA), 22C3 (DAKO), SP263 (Ventana, Tucson, Arizona, USA), SP142 (Ventana)]. Automated pre-treatment was performed at pH 6 for 28–8 / 22C3, and pH 9 for SP142 / SP263. Primary monoclonal antibodies were incubated for 30 min at room temperature and visualized using the appropriate DAB-based detection kits and haematoxylin counterstains (Agilent/DAKO Envision system autostainer plus, Ventana Benchmark Ultra). For lab developed immunohistochemical tests negative controls were run by omitting the primary antibody for both pH conditions compared to positive controls (see Additional files 1 and 2). PD-L1 expression was determined for tumour cells (TP-Score), immune cells (IC-Score) and combined (CPS, combined positivity score) regardless of the staining intensity as follows: TPS/ Cologne Score: 0 = 0 < 1%, 1 = 1 - < 5%, 2 = 5 - < 10%, 3 = 10 - < 25%, 4 = 25 - < 50%, 5 = > 50% [11], IC/Immune cell Score: 0 = < 1%, 1 = 1- < 5%, 2 = 5- < 10%, 3 = > 10% [20], and the combined positivity score (CPS) given by summing the number of PD-L1–stained cells (tumour cells, lymphocytes, macrophages) and dividing the result by the total number of viable tumour cells, multiplied by 100 [21]. Cores with staining artefacts or damage were excluded. Scoring was performed by two independent investigators (RM and NTG). Inter-observer discrepancies regarding the percentage of positivity or scoring were discussed and a consensus was found.

### Statistical analysis

Statistical analyses were performed using SPSS 25.0 (SPSS, Chicago, IL, USA) and GraphPad Prism 5.0 (GraphPad Software Inc., La Jolla, CA). Differences were considered statistically significant if the two-sided *p*-values were equal or below 5% (≤0.05). The non-parametric Mann-Whitney U-test was used in order to compare two groups*.* In case of more than two groups the non-parametric Dunn’s multiple comparison test was used. Correlation analysis was performed by calculating a non-parametric *Spearman’s rank* correlation coefficient.

## Results

### Staining results of four different PD-L1 antibodies in pure SCC and mixed UC/SCC

**Fig. 1:**
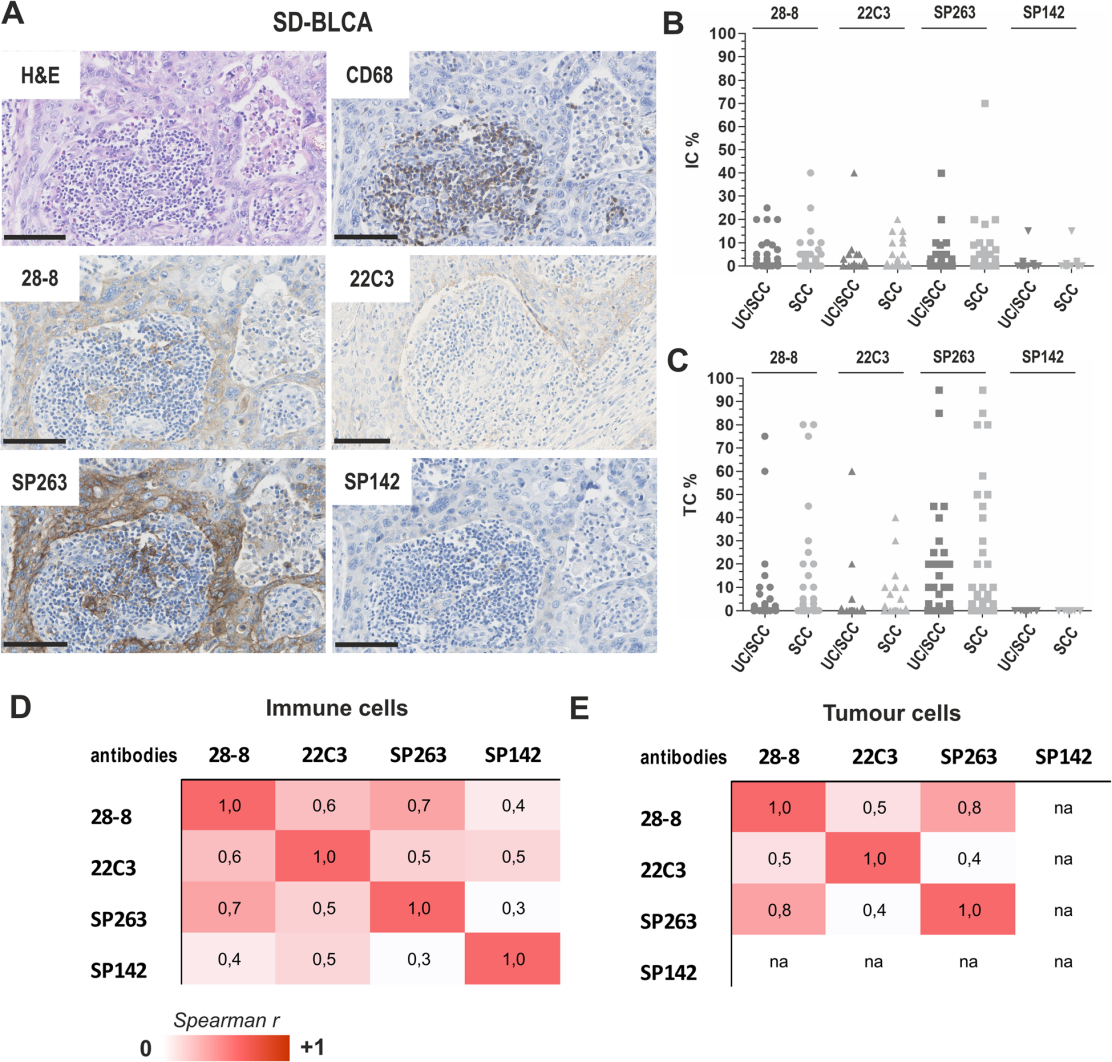
PD-L1 protein expression in squamous differentiated bladder cancer (SD-BLCA). **a** Immunohistochemical PD-L1 staining is shown for representative tissue cores illustrating both immune cells (IC) and tumour cells (TC) by applying four different antibodies: DAKO 28–8, DAKO 22C3, Ventana SP263 and Ventana SP142. Squamous tumour components are histologically shown by H&E staining. CD68 staining highlights macrophages. Black scale bar: 100 μM**.** Please note: Due to tissue loss during the first immunohistochemical staining with the 22C3 antibody deeper tissue sections of the patient’s FFPE material were used which show slight differences in histology. **b-c** Scatter plot graphs show overall distribution of PD-L1 positive areas of IC and TC for mixed (UC/SCC) and pure squamous cancers (SCC). **d-e** Spearman correlation analysis demonstrating inter-assay heterogeneity for IC (**d**) and TC (**e**)

108 squamous differentiated bladder cancers comprising 45 mixed UC/SCC and 63 SCC (for cohort characteristics see Table 1) were immunohistochemically stained with four different anti-PD-L1 antibodies, i.e. the Dako 28–8 and 22C3 and the Ventana SP263 and SP142 (Fig. 1a). PD-L1 antibodies showed variable staining results for both immune (IC) and tumour cells (TPS) in UC/SCC and SCC (Fig. 1b and c). In mixed UC/SCC positive staining was determined for immune cells (IC-score ≥ 1) in 48.8% (28–8; 21/43), 20.5% (22C3; 9/44), 58.1% (SP263; 25/43) and 11.1% (SP142; 5/45) (Fig. 1b). Tumour cells showed PD-L1 expression (TPS ≥1) in 39.5% (28–8; 17/43), 11.3% (22C3; 5/44), 51.2% (SP263; 22/43) and 0% (SP142, 0/45) (Fig. 1c). In pure SCC we observed IC-scores ≥1 in 39.7% (28–8; 25/63), 31.1% (22C3; 19/61), 61.9% (SP263; 39/63) and 4.8% (SP142; 3/63) (Fig. 1b). TPS ≥1 was found in 28.6% (28–8; 18/63), 16.4% (22C3; 10/61), 47.6% (SP263; 30/63) and 0% (SP142, 0/63) (Fig. 1c). Non-parametric Spearman-rank correlation significantly demonstrated a high similarity in PD-L1 staining of SP263 and 28–8 antibodies for IC (r: 0.734, *p* < 0.001) and TPS (r: 0.773, *p* < 0.001) (Fig. 1d-e). Including 22C3 assay, inter-assay correlation (*p* < 0.001) ranged between 0.532 (SP263) and 0.617 (28–8) for IC and 0.409 (SP263) and 0.527 (28–8) for TPS. The SP142 assay showed weakest overlap, i.e. the Spearman correlation coefficient ranged between 0.332 and 0.509 for IC, while for TPS no correlation was accessible (no tumour cell staining). Furthermore, evidence for different PD-L1 expression between mixed UC/SCC and pure SCC was not observed. For detailed scoring results see Table 2.

**Table 1:** Clinico-pathological parameters of 108 SD-BLCA samples analysed in this study by immunohistochemistry

	Categorisation	n^a^ analysable	%
***Parameter:***			
Age at diagnosis:	median: 67.5 years		
	(range 33–88)		
	< 67.5 years	53	49.1
	≥67.5 years	53	49.1
	na	2	1.8
Gender	male	52	48.1
	female	53	49.1
	na	3	2.8
Tumour subtype	UC/SCC	45	41.7
	SCC	63	58.3
Histological tumor grade^b^	G1	1	0.9
	G2	29	26.9
	G3	73	67.6
	G4	1	0.9
	na	4	3.7
Tumour stage^c^	pTx	6	5.6
	pT1	1	0.9
	pT2	13	12.1
	pT3	70	64.8
	pT4	18	16.6
Lymph node status	negative (pN0)	66	61.0
	positive (pN1 + pN2)	21	19.5
	na	21	19.5

^a^Only patients with primary bladder cancer were included; ^b^According to WHO 1973 classification; ^c^According to UICC TNM classification 8th edition; na: not available

**Table 2:** PD-L1 expression in mixed UC/SCC and pure SCC

	pure SCC				mixed UC/SCC			
Scores	28–8	22C3	SP 263	SP142	28–8	22C3	SP 263	SP142
**TC (%) 0**	45	51	33	63	26	39	21	43
**TC (%) 1**	5	2	10	0	9	2	3	0
**TC (%) 2**	2	2	4	0	2	1	1	0
**TC (%) 3**	5	4	5	0	4	1	10	0
**TC (%) 4**	3	2	6	0	0	0	6	0
**TC (%) 5**	3	0	5	0	2	1	2	0
**IC (%) 0**	38	42	24	58	22	35	18	40
**IC (%) 1**	8	8	21	4	12	2	11	3
**IC (%) 2**	14	4	14	0	5	6	12	1
**IC (%) 3**	3	7	4	1	4	1	2	1
**CPS < 1**	18	39	11	49	13	33	8	36
**CPS 1–9**	32	10	32	13	20	8	15	5
**CPS ≥ 10**	13	12	20	1	10	3	20	1

*TC* tumour cell area (%), *IC* immune cell area (%), *CPS* combined positivity score

### Therapeutic implications of staining results

**Fig. 2:**
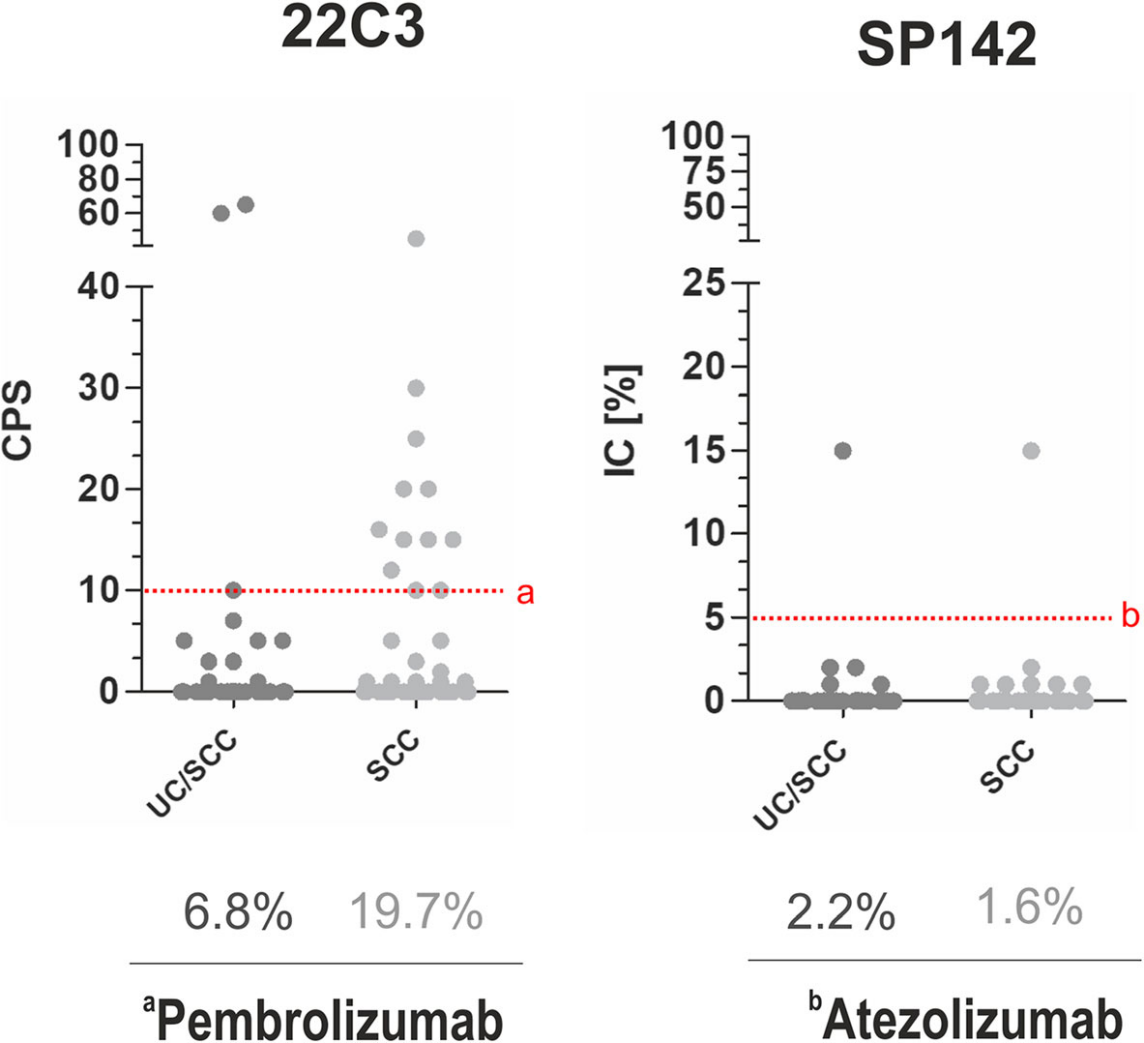
Therapeutic implications of used PD-L1 antibodies in SD-BLCA according to FDA-approved guidelines for first line therapy in bladder cancer. Scatter plots represent CPS and IC for DAKO 22C3 and Ventana SP142, respectively. *Red dotted line*: drug-related cut-off values. *Below:* Percentages of patients with putative choice of first line therapy with pembrolizumab (**a**) and atezolizumab (**b**)

According to the current FDA-approved guidelines for first line therapy of bladder cancer with pembrolizumab (CPS ≥10) and atezolizumab (IC-score ≥ 2 / IC ≥ 5%), we determined patients with putative choice of first line ICI therapy, overall ranging between 2 and 20% in SD-BLCA (Fig. 2). For pembrolizumab, a 22C3 CPS cut-off ≥10 indicates putative therapy access in 7% of patients with mixed UC/SCC and in 20% of SCC patients. SP142 completely failed to hold clinical significance. By focusing on the European Medicines Agency (EMA) guidelines according to which strict/mandatory PD-L1 companion diagnostics assay settings are not required by now, up to 47% of UC/SCC and up to 32% of SCC patients would be eligible for first line PD-L1 checkpoint inhibitors (Table 3).

**Table 3:** Frequencies according to EMA guidelines for 1st line ICI therapy of urothelial cancers

Scores	28–8	22c3	SP263	SP142	28–8	22c3	SP263	SP142
**IC ≥ 5%**^**a**^	(17/63) 27%	(11/61) 18%	(18/63) 29%	(1/63) 2%	(10/43) 23%	(7/44) 16%	(14/43) 33%	(1/45) 2%
**CPS ≥ 10**^**b**^	(13/63) 21%	(12/61) 20%	(20/63) 32%	(1/63) 2%	(10/43) 23%	(3/44) 7%	(20/43) 47%	(1/45) 2%

^a^atezolizumab; ^b^pembrolizumab

### Clinical example for immune checkpoint inhibitor treatment in a SD-BLCA patient

**Fig. 3:**
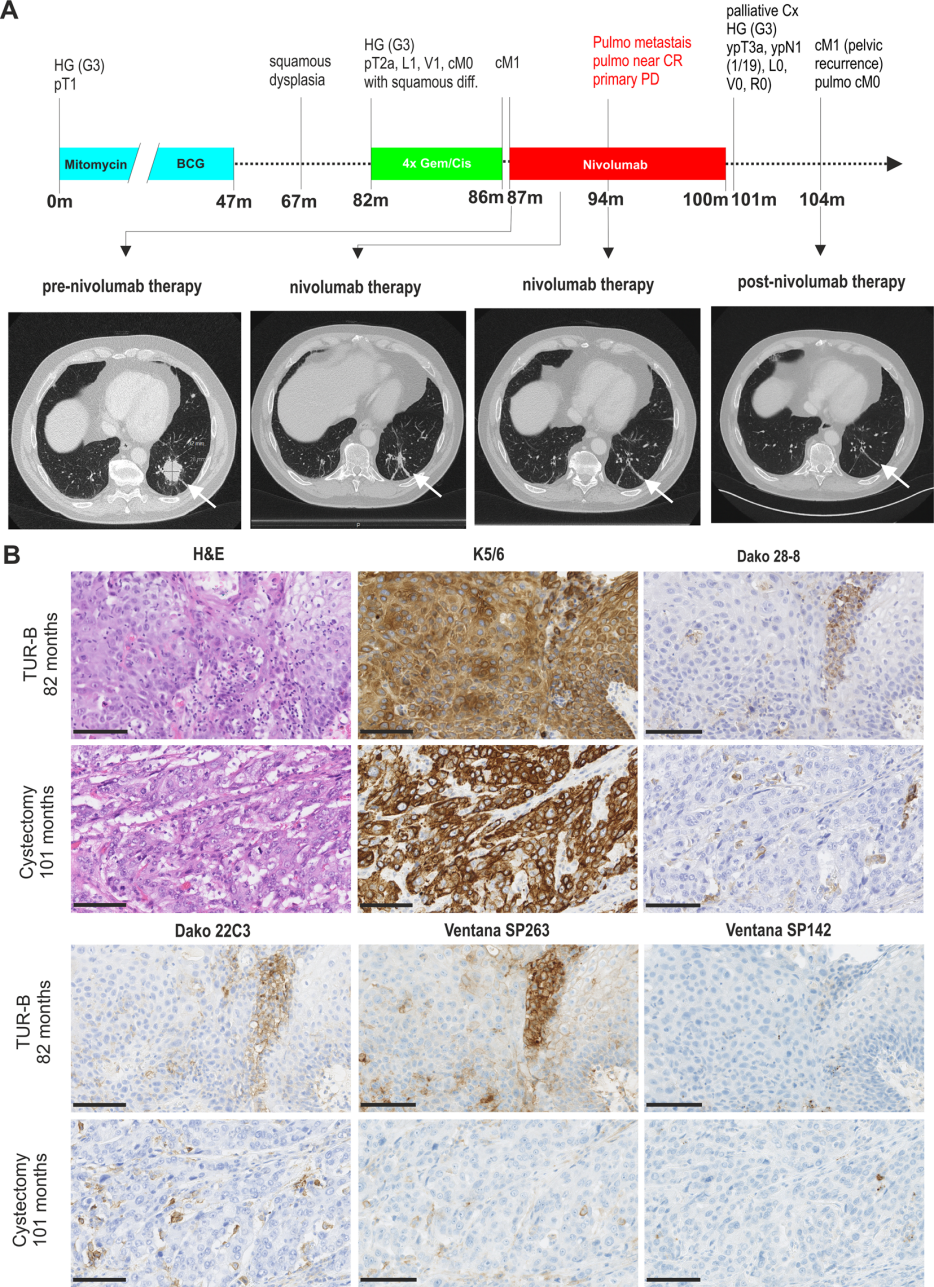
SD-BLCA index patient treated with nivolumab in a second line therapy. **a***Upper:* History time line of index patient illustrating the diagnostic and therapeutic management over 140 months since first diagnosis. *Below*: CT images of the index patient show pulmonary metastasis size (white arrow) at different therapy time points. **b** Immunohistochemical PD-L1 staining of primary tumour lesions of the index patients derived from tissues removed before and after nivolumab treatment is shown. Squamous components are histologically shown by H&E staining and highlighted by K5/6 staining. PD-L1 expression was determined in both tumour cells (TC) and immune cells (IC) by using four different antibodies: DAKO 28–8, DAKO 22C3, Ventana SP263 and Ventana SP142. Black scale bar: 100 μM**.***HG*: high grade; *pT*: pathological tumour stage; *L*: invasion into lymphatic vessels; *V*: invasion into vein; *R*: the completeness of the operation; *pN*: pathological degree of spread to regional lymph nodes; *cM*: clinical metastasis; *CR*: complete response, *PD*: progressive disease; *Cx*: cystectomy; *TUR-B*: transurethral resection of the bladder; m: months

A 62-year old male patient was first diagnosed with a high grade (G3) pT1 urothelial carcinoma of the urinary bladder in 2009 and his medical history is shown in Fig. 3a. Subsequently post resection and surveillance biopsy showed no evidence of malignancy but keratinizing squamous metaplasia of the urothelium. He received mitomycin-instillation and following BCG maintenance therapy for 47 months. In 2015 a TUR-B sample displayed moderate to severe squamous epithelial dysplasia, but there was no evidence for invasive carcinoma. 15 months later a subsequent invasive urothelial carcinoma (high grade (G3), min. pT2a, L1, V1) with substantial squamous differentiation without radiological evidence of metastasis was diagnosed. He received 4 cycles gemcitabine/cisplatin chemotherapy. Treatment was switched to second line palliative checkpoint inhibitor therapy with nivolumab due to progressive pulmonary metastasis. CT-staging monitoring is shown before, during and after immune checkpoint inhibitor treatment providing evidence of a partial response, i.e. long-lasting near-complete response of the pulmonary metastasis (Fig. 3b) and initial response (over the first 3 months upon nivolumab treatment) of the local tumour, but thereafter progressive disease (data not shown). Histological documentation and subsequent immunohistochemical PD-L1 staining of tissue samples at two different time points (before and after nivolumab therapy) confirmed squamous differentiation with a proportion of 80 and 30% of the primary tumour lesion, respectively. Biopsies from the pulmonary metastatic site had not been taken. PD-L1 expression was demonstrated for immune cells while it was barely detectable in tumour cells (Fig. 3c). In fact, PD-L1 expression was shown for 28–8 in 30%, for 22C3 in 30% for SP263 in 25% and for SP142 in 7% of IC before nivolumab treatment. In parallel, 1% of tumour cells were stained positively by applying DAKO 28–8 and 22C3 while Ventana SP263 led to 7% PD-L1 staining in tumour cells. Tumour cells were negative using SP142 (Table 4). Interestingly, at the point where ICI therapy has been completed, immune cells showed reduced PD-L1 expression varying between 7 and 10%. PD-L1 staining was not observed in tumour cells (Table 4). Significant differences in PD-L1 expression between urothelial and squamous differentiated tumour areas were not observed.

**Table 4:** TC, IC and CPS of tissue samples of the index patients before and after nivolumab therapy

Type	squamous component	28–8			22C3			SP263			SP142		
		IC%	TC%	CPS	IC%	TC%	CPS	IC%	TC%	CPS	IC%	TC%	CPS
TUR-B 82 months	80%	30	1	30	30	1	30	25	7	30	7	0	7
Cystx 101 months	15%	10	0	10	10	0	10	10	0	10–12	7	0	7

## Discussion

So far, the clinical management of patients with squamous differentiated bladder cancer is limited by the choice of effective (neo) adjuvant therapies [13–15]. The five year survival rate is worse varying between 16 and 48% [22, 23]. A ray of hope might be immunotherapy by PD1/PD-L1 checkpoint inhibitors which recently revolutionized the therapeutic landscape of various cancers including urothelial cancer [24]. In urothelial cancers efficacy of different ICIs have been assessed in clinical trials during the last years [7]. For instance, the Keynote-045 trial demonstrated a clinical benefit of pembrolizumab over chemotherapy for efficacy and safety upon treatment of locally advanced/metastatic, platinum-refractory urothelial tumours [25]. Meanwhile two ICI agents, i.e. pembrolizumab and atezolizumab, have been approved by the FDA and EMA for first-line therapy of platinum-ineligible patients with PD-L1 expression as specified by scoring algorithms [8, 9]. Accumulating studies also indicate strong PD-L1 expression in squamous tumors of the urinary bladder [17–19], but underlying retrospective cohorts are less suitable to assess the additive value of ICI therapies in SCC disease management: The patient cohort analyzed by Owyong and colleagues comprised mainly Schistosomiasis-associated SCC [17], while the publications by both Reis et al. and Udager et al. lack sufficient SCC sample numbers [18, 19]. Moreover, all studies were characterized by the absence of a clinical setting.

In the presented study, we now provide evidence for suitable ICI treatment of squamous bladder cancer by analysing both PD-L1 staining of a larger retrospective cohort of 108 SD-BLCA samples and of a SD-BLCA index patient whose pulmonary metastasis showed complete response upon nivolumab treatment. In concordance with the current FDA/EMA guidelines of urothelial cancers calling for PD-L1 positivity to protect from side effects [8, 9, 26], we revealed frequent PD-L1 expression in squamous bladder tumours up to 62% for immune and up to 52% for tumour cells. These findings, based on four different antibodies (DAKO 28–8, DAKO 22C3, Ventana SP263 and Ventana SP142), confirmed the data of the recent publications [17–19] and are comparable with studies of urothelial cancer [10]. So far, ICI treatment is an integral part of the therapy of squamous cancers in other organs like the lung and head and neck: The Checkmate-017 study revealed an improved overall survival (OS) and a favourable safety profile for nivolumab compared to docetaxel in patients with pre-treated squamous NSCLC [27]. The Keynote-407 study showed clinical significance of combined pembrolizumab treatment with chemotherapy in patients with metastatic squamous NSCLC [28]. In HNSCC the Keynote-012 study demonstrated clinically significant activity in patients with pre-treated tumors for pembrolizumab irrespective of human papillomavirus (HPV) status [29].

However, different PD-L1 antibodies, associated immunohistochemical assays and scoring algorithms, still challenge a robust selection of patients who will benefit from ICI treatment. In line with previous studies in urothelial cancer [10, 12], we also confirmed a substantial inter-assay heterogeneity of PD-L1 expression in squamous bladder cancer. Scheel et al. reported, that the four PD-L1 assays do not show comparable staining patterns in NSCLC [11]. The Blueprint PD-L1 Immunohistochemistry (IHC) Assay Comparison Project also studied the performance of the four PD-L1 IHC assays (22C3, 28–8, SP142, and SP263) in NSCLC, and found -very similar to our results- an analytical comparability of 22C3, 28–8, and SP263 whereas the SP142 assay showed lowest levels of correlation [30]. Hirsch and colleagues concluded that despite similar analytical performance of PD-L1 expression, interchanging assays and cut-offs would lead to “misclassification” of PD-L1 status for a substantial amount of patients. As a consequence, different patient numbers would be eligible for first line therapy with PD-L1 checkpoint inhibitors but still without clear evidence which staining results and cut-off levels really predict therapy response. For urothelial bladder cancer, the clinical consequences with substantial amounts of discordant classifications due to inter-assay and especially inter-algorithm variability, i.e. nearly 50% discordances between eligibility for first line treatment with atezolizumab or pembrolizumab, have been reported previously [10]. In clinical trials, for instance, the objective response rate (ORR) of urothelial cancer patients with nivolumab treatment did not significantly differ between PD-L1 positive (> 1%) and PD-L1 negative tumors (< 1%) (Checkmate-032 study) [31]. Beyond that the reliability of PD-L1 assays to predict ICI response is reduced by various aspects such as non-immunity dependent upregulation of PD-L1 expression (e.g. via *PTEN*) [32] or intratumoral heterogeneity and dynamic alteration by treatment and cancer progression [24]. In turn, higher ORR have been shown to be associated with increased PD-L1 expression also in urothelial cancer [33]. In this Keynote phase 2 study the subgroup of bladder cancer patients with PD-L1 expression above a cut-off ≥10% showed highest ORR upon pembrolizumab treatment. PD-L1 expression, as revealed in our cohort of squamous bladder cancers, may thereof be suitable to facilitate patient selection for ICI therapies.

This notion is supported by the here presented index patient with a squamous bladder cancer demonstrating partial therapy success upon ICI treatment. Prior to nivolumab treatment, the primary tumour exhibited a substantial percentage of squamous differentiation (80%) with strong PD-L1 expression. Upon ICI treatment the primary tumour showed clinically only short response but thereafter local progress was observed. Interestingly, after nivolumab therapy completion the progressive tumour lesion (< 50% squamous component) was characterized by reduced PD-L1 positivity. Of clinical significance, the pulmonary metastasis showed long-lasting response without any evidence of harmful side effects.

## Conclusion

Our data reveal strong PD-L1 expression in squamous differentiated bladder cancers comparable with urothelial cancer whose disease management has been successfully improved by ICI therapy. Considering the encouraging clinical data of our index patient we propose to consider treatment of ICI also for both mixed and pure SCC of the urinary bladder. However, according to the tumour and inter-assay heterogeneity of PD-L1 expression, the utility of given scoring algorithms for robust patients’ therapy selection remains questionable and should be considered in future study designs.

## Supplementary information

**Additional file 1: Figure S1.** Lab developed immunohistochemistry: HE staining and negative controls are shown for pH 6 as well as pH 9 by omitting the primary antibody.

**Additional file 2: Figure S2.** Tonsil tissue used as positive control. HE staining and PDL1 immunohistochemistry using different antibody clones: DAKO 28–8, DAKO 22C3, Ventana SP263 and Ventana SP142.

## Data Availability

The datasets used and/or analysed during the current study are available from the corresponding author on reasonable request.

## Abbreviations

CPS: Combined positive score
EMA: European Medicines Agency
FDA: Food and Drug Administration
FFPE: Formalin-fixed paraffin-embedded
IC-score: Immune cell score
ICI: Immune checkpoint inhibitors
ORR: Objective response rate
PD-L1: Programmed death-ligand 1
SD-BLCA: Squamous differentiated bladder cancer
SCC: Squamous cell carcinoma
TPS: Tumour proportion score
TMA: Tissue microarray
UC/SCC: Urothelial carcinoma with squamous differentiation
