# Supplementary material for: Therapeutic implications of PD-L1 expression in bladder cancer with squamous differentiation
Source: BMC Cancer. 2020 Mar 18;20:230. doi: 10.1186/s12885-020-06727-2 (PMC7079494; doi:10.1186/s12885-020-06727-2)
Supplement: Supplementary file 1 — Additional file 1: Figure S1. Lab developed immunohistochemistry: HE staining and negative controls are shown for pH 6 as well as pH 9 by omitting the primary antibody. [file 12885_2020_6727_MOESM1_ESM.docx]

**Additional file 1**


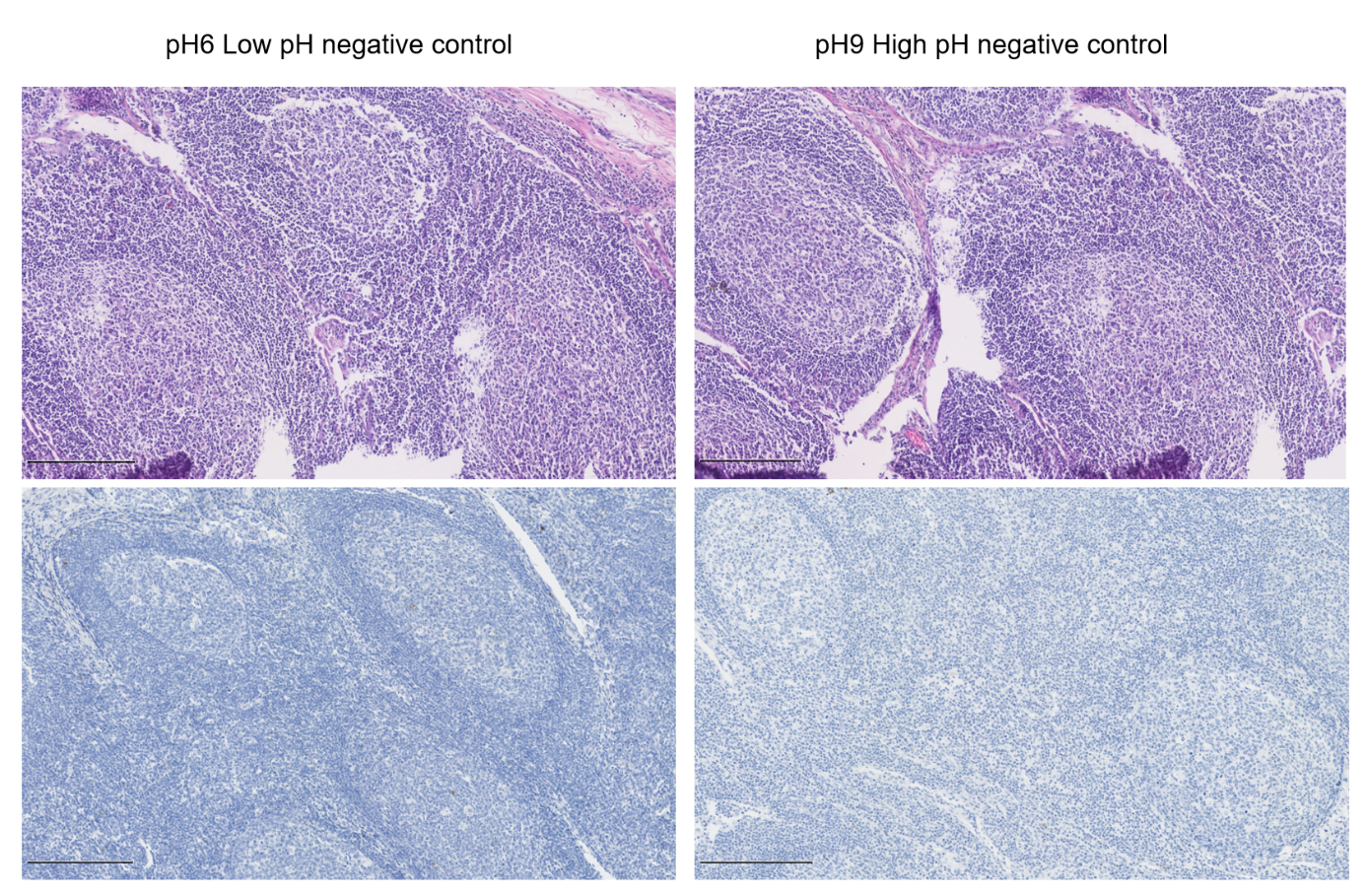


**Supplemental Figure 1:** Lab developed immunohistochemistry: HE staining and negative controls are shown for pH6 (left) as well as pH9 (right) by omitting the primary antibody (Black scale bar: 200 µM**).**
