# Supplementary material for: Therapeutic implications of PD-L1 expression in bladder cancer with squamous differentiation
Source: BMC Cancer. 2020 Mar 18;20:230. doi: 10.1186/s12885-020-06727-2 (PMC7079494; doi:10.1186/s12885-020-06727-2)
Supplement: Supplementary file 2 — Additional file 2: Figure S2. Tonsil tissue used as positive control. HE staining and PDL1 immunohistochemistry using different antibody clones: DAKO 28–8, DAKO 22C3, Ventana SP263 and Ventana SP142. [file 12885_2020_6727_MOESM2_ESM.docx]

**Additional file 2**


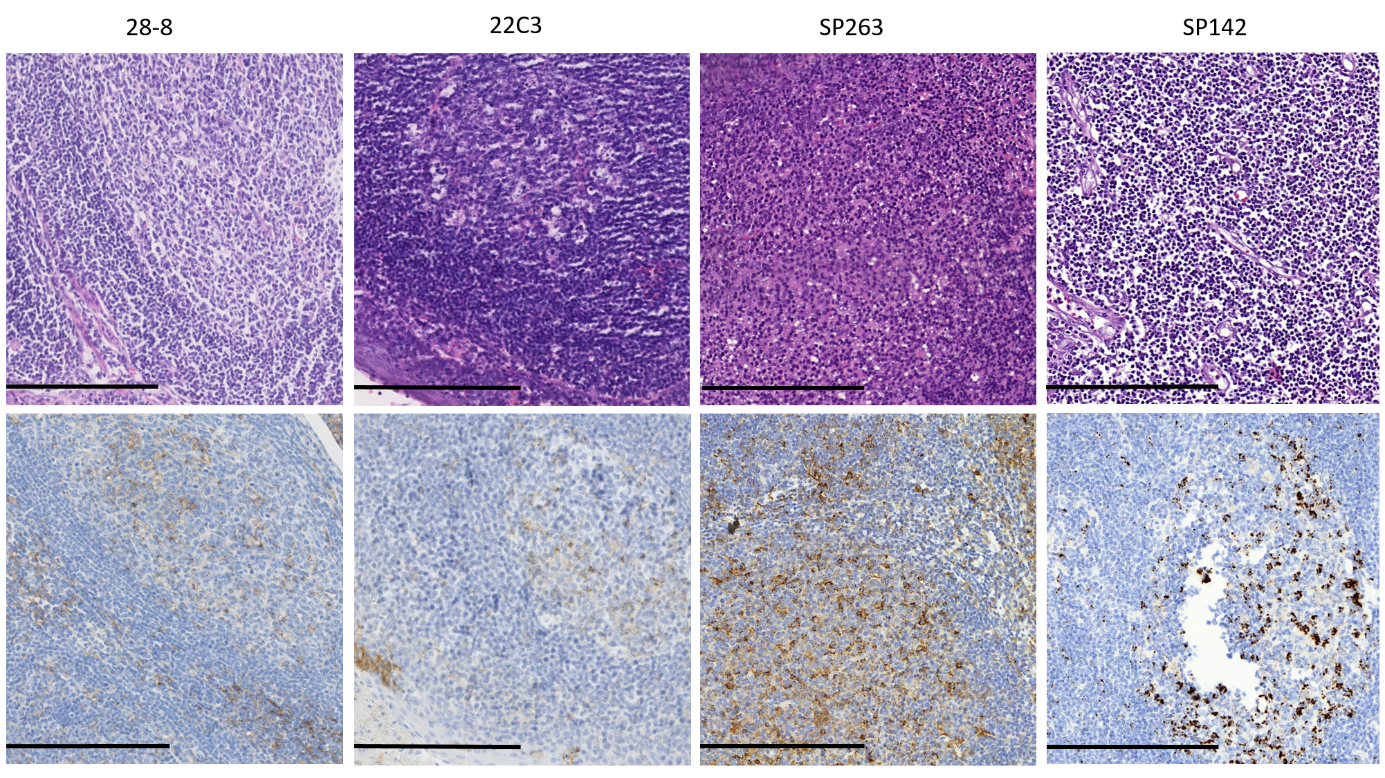


**Supplemental Figure 2:** Tonsil tissue used as positive control. HE staining and four different antibodies: DAKO 28-8, DAKO 22C3, Ventana SP263 and Ventana SP142 (Black scale bar: 200 µM**).**
